# Redox-Sensitive Nanocomplex for Targeted Delivery of Melittin

**DOI:** 10.3390/toxins12090582

**Published:** 2020-09-10

**Authors:** Bei Cheng, Peisheng Xu

**Affiliations:** Department of Discovery and Biomedical Sciences, College of Pharmacy, University of South Carolina, 715 Sumter, Columbia, SC 29208, USA; beicheng2012@gmail.com

**Keywords:** nanocomplex, targeted delivery, melittin, redox sensitive, cancer

## Abstract

Although peptide therapeutics have been explored for decades, the successful delivery of potent peptides in vitro and in vivo remains challenging due to the poor stability, low cell permeability, and off-target effects. We developed a redox sensitive polymer-based nanocomplex which can efficiently and stably deliver the peptide drug melittin for cancer therapy. The nanocomplex selectively targets cancer cells through lactobionic acid mediated endocytosis and releases melittin intracellularly upon the trigger of elevated redox potential. In vivo study proved that the targeted nanocomplex shows excellent potency in inhibiting tumor growth in a xenograft colon cancer mouse model. Thus, the polymer/melittin nanocomplexes will provide a new approach for melittin based cancer therapy.

## 1. Introduction

Peptide-based drugs, which usually have less than 50 amino acids, hold a high potential for treating various diseases. Furthermore, some peptide-based drugs have successfully entered the market, such as cyclosporine, which is routinely used after organ transplantation to reduce the activity of the immune system [1]. Some peptides can also be utilized as a cytosolic agent. One of the most well-established peptides is luteinizing hormone-releasing hormone agonists for the combination treatment of prostate cancer combined with radiation or chemotherapy [2,3]. The last decade has witnessed the exponential growth of many peptide drugs entering clinical trials [4]. Specifically, peptides hold many advantages for anticancer medications due to their high potency and a broad range of targets. However, many hurdles, including vulnerability toward proteolytic digestion and side effects due to off-targeting, have yet to be overcome [5,6,7]. For instance, the available cyclosporine preparations showed a low bioavailability, high variation between patients, and the inability to obtain both effective and safe doses [1].

Recently, melittin, a natural peptide from bee venom composed of 26 amino acids, has attracted a lot of attention due to its anti-arthritis, anti-inflammatory, anticancer, and neuroprotective properties [8,9,10,11,12,13]. Melittin self-assembles into toroidal structures and induces the formation of pores on the cell membrane, which ultimately results in cell death [14,15]. This unique cytolytic feature makes it impossible for cancer cells to develop drug resistance. Due to the same reason, the non-specific cytolytic activity of melittin could result in severe off-target effects, such as hemolysis (lysis of red blood cells), when administrated intravenously. To make melittin druggable and safer, many approaches have been explored through environmental liable modification [16,17] or nanoencapsulation [18,19,20]. Previously, our group utilized a dual-secured “nanobee” technology with the help of succinic anhydride-modified glycol chitosan (SAMGC), and disulfide bonds successfully increased the therapeutic window of melittin to 10 µM [21]. The positively charged melittin forms nanocomplexes with a negatively charged SAMGC through the electrostatic effect, while the disulfide bonds ensure that the encapsulated melittin can only be released inside the cytosol, where it has elevated glutathione (GSH), to prevent the occurrence of hemolysis.

Our group recently developed a poly[(2-(pyridin-2-yldisulfanyl) ethyl acrylate)-co-[poly(ethylene glycol)]] (PDAPEG) polymer [22,23,24], which can be easily modified by thiol-disulfide exchange reaction. Thanks to this nature, a serial of zwitterionic polymers with different isoelectric points (IEPs) could be produced by simply tuning the ratio of the introduced NH_2_ and COOH groups. Since the intracellular environment usually contains a 1000-fold higher level of GSH than that in the extracellular milieus [25], the high redox potential can be utilized to trigger the release of peptides by breaking the disulfide bonds.

To improve the specificity for cancer targeted delivery, various cancer cell-related receptors have been explored. Among them, a β-D-galactose receptor, also known as ASGPR1, has been extensively investigated. It was revealed that ASGPR1 receptor is expressed in multiple types of cancer, including ovarian cancer, liver cancer, breast cancer, and colon cancer [26,27,28]. Therefore, many ligands for ASGRP1 receptor have been developed, including lactobionic acid (LBA), for cancer targeted therapy [29]. Colon cancer can develop acquired multidrug resistance during conventional treatment [30] and has been validated for LBA targeting in our previous research.

**Figure 1:**
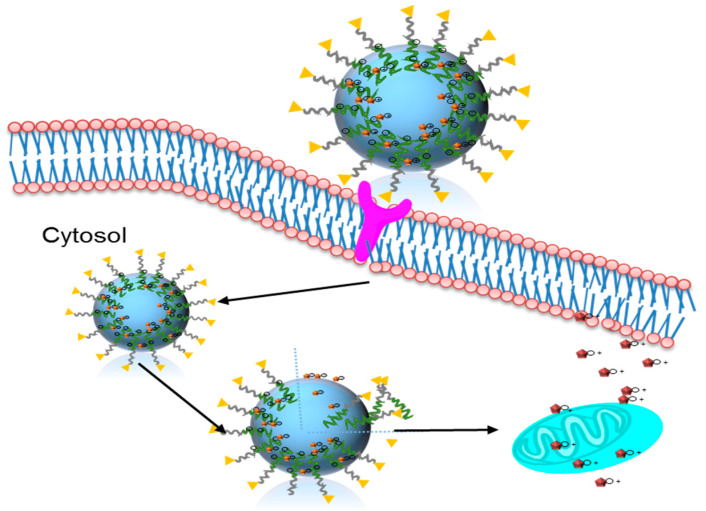
Delivery pathway of targeted melittin nanocomplex for cancer therapy.

Herein, we developed a redox responsive, ASGRP targeted polymer/melittin nanocomplex, for the treatment of colon cancer. The negatively charged PDAPEG polymer derivative forms complex melittin through electrostatic force. The nanocomplexes enter cancer cells through LBA mediated endocytosis, subsequently dissociate due to the cleavage of disulfide bonds, and release melittin intracellularly to kill cancer cells (Figure 1). Since there is no premature release of melittin before it enters cancer cells, we believe this nanocomplex can translate targeted melittin therapy to a clinically promising treatment with high efficiency and low side effects.

## 2. Results and Discussion

### 2.1. Design and Synthesis of Polymer LBA-PDAPEG

In our recent work, we prepared an environment-sensitive peptide delivery system, dual secured nano-sting through the combination of a zwitterionic glycol chitosan and disulfide bond [21]. In that design, melittin was successfully delivered into cytosol and released under the acidic pH and high redox environment. In this study, we aimed to deliver melittin through a redox sensitive polymer PDAPEG, as shown in Figure 1.

**Figure 2:**
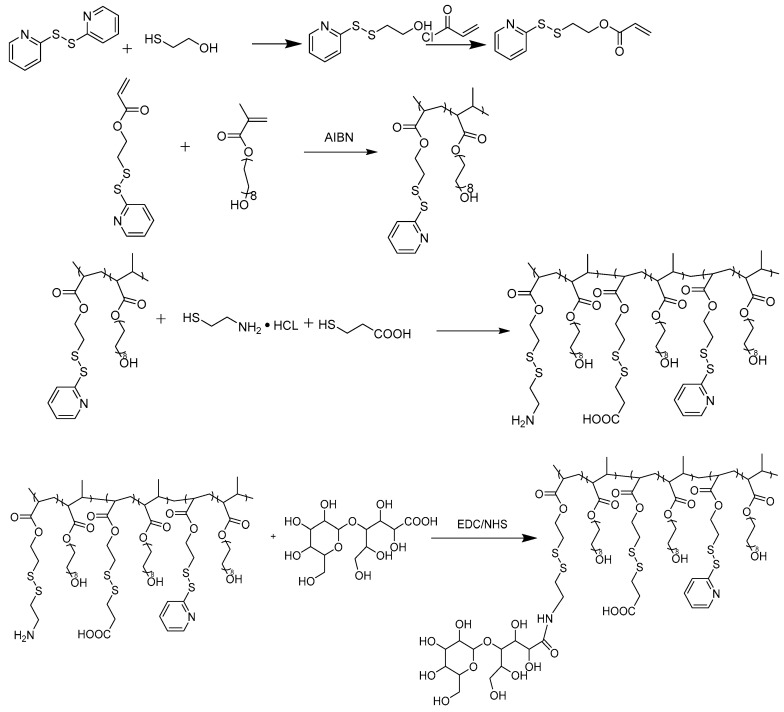
The synthesis scheme of LBA-PDAPEG.

**Figure 3:**
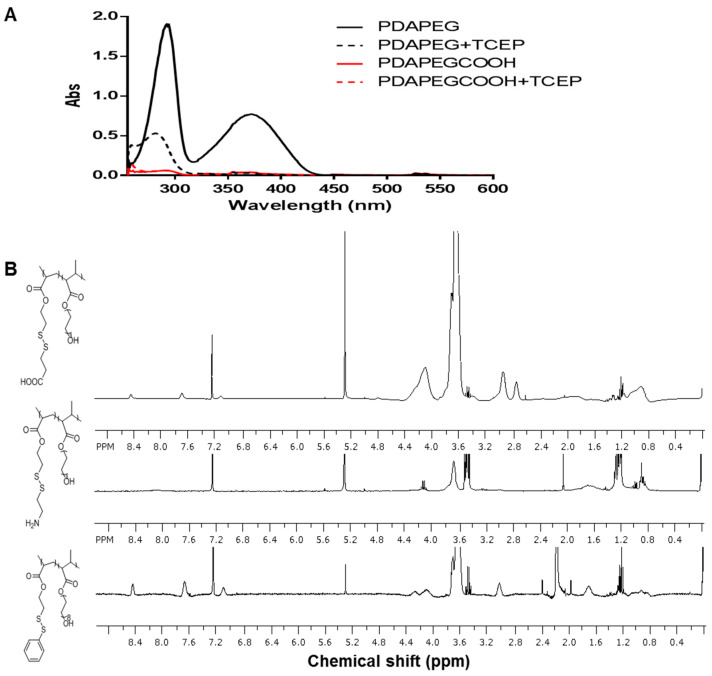
(**A**). UV–Vis spectrum of polymer PDAPEG or PDAPEG-COOH when co-incubating with 10 mM TCEP. The appeared peak at 375 nm indicates the existence of pyridinethiol in polymer; (**B**). ^1^H-NMR of polymer PDAPEG-COOH, PDAPEG-NH_2_, and PDAPEG in CDCl_3_.

The synthesis of LBA-PDAPEG is described in Figure 2. To introduce carboxylic acid groups and amine groups into the PDA-PEG polymer, the pyridine ring was replaced completely by cysteamine or 3-mercaptopropionic acid through thiol disulfide exchange reaction. The complete of the reaction was confirmed by UV–vis and ^1^H-NMR. The signature peak of 2-pyridinethiol at 375 nm after cleavage by tris(2-carboxyethyl)phosphine (TCEP) in the UV–vis spectrum disappeared after the thiol disulfide exchange in Figure 3A. The disappeared protons in pyridine rings (8.46, 7.69, 7.11 ppm) in the ^1^H-NMR spectrum, as shown in Figure 3B, support the conclusion. Successful conjugation of 3-mercaptopropionic acid was characterized by the –CH_2_CH_2_– protons (2.95, 2.74 ppm).

**Figure 4:**
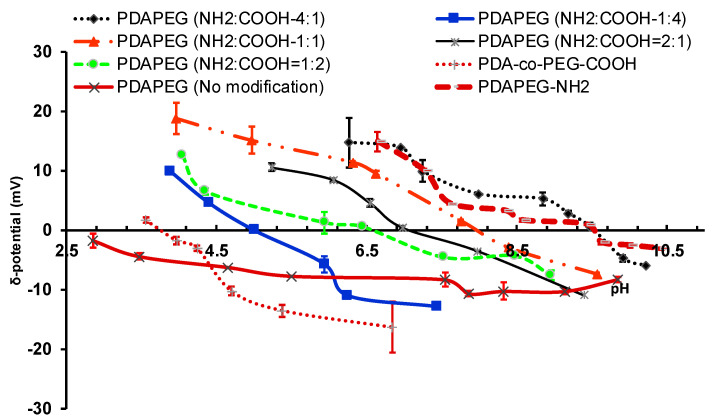
The surface charges of PDAPEG-NH2/PDAPEG-COOH at different amine to carboxylate ratio responsive to environmental pH.

The composition of amine to carboxylic acid group determines the isoelectric point (IEP) of polymer, which can be tuned by varying the feeding ratio of cysteamine to 3-mercaptopropionic acid. The IEP points of polymers ranged from 9.5 to 5.0 when the cysteamine to 3-mercaptopropionic acid was fed from 4/1 to 1/4 (Figure 4). The wide range of tunable surface charge of the polymers would allow for the binding of peptides with different IEPs. The gentle electrostatic force between polymer and peptide would prevent the potential denature of peptide if prepared with the traditional encapsulation method accompanied with high intensive mechanical force or heat. In our study, we used a PDAPEG derivative with a surface charge around −11 mV at the pH of 7.4 for nanocomplex fabrication. The targeting moiety of LBA was conjugated to the amine group of through N-hydroxysuccinimide (NHS) and 1-thyl-3-(3-(dimethylamino)propyl) carbodiimide (EDC) reaction. The conjugation was quantitatively determined by the reduction of amine amount through a 2,4,6-trinitrobenzene sulfonic acid (TNBSA) assay. The unmodified PDAPEG was determined to have 500 nmol pyridine rings per 1 mg polymer with the co-incubation with 10 mM dithiothreitol (DTT). After the modification of both by cysteamine and mercaptopropionic acid, the total amine amount was increased to 40.6 nmol per mg polymer. After the conjugation of LBA, the available amine was decreased to 32.5 nmol per mg polymer, which indicates a successful conjugation of LBA.

### 2.2. Particle Characterization

**Figure 5:**
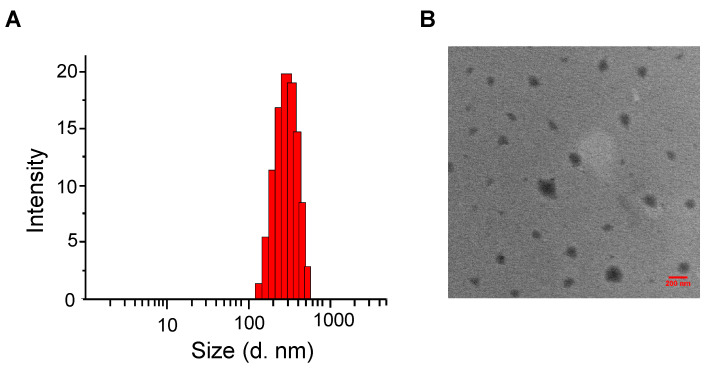
Characterization of the nanocomplexes. Hydrodynamic size of nanocomplex in TBS buffer measured by dynamic light scattering (**A**) and TEM image of nanocomplex (**B**). Scale bar in (**B**) is 200 nm.

Through electrostatic complexation, a negatively charged PDAPEG derivative formed with complexed positively charged peptide melittin at physiology pH and further condensed it. After the formation of complex, melittin was entrapped within the network of nano-structure and was protected through the outlayer hydration of PEG. The nanocomplex showed a particle hydrodynamic size of 357 ± 30 nm (Figure 5). They were almost spherical according to transmittance electron microscopy. In addition, the surface charge of nanocomplex dropped from −11.8 ± 0.64 to −4.24 ± 0.21 mV also indicates the successful complexation with melittin. This proper size would allow for the passive transportation of nanoparticles entering cancer cells through enhanced permeability and retention effect [31].

### 2.3. High Redox Potential Environment Could Trigger the Release of Melittin

**Figure 6:**
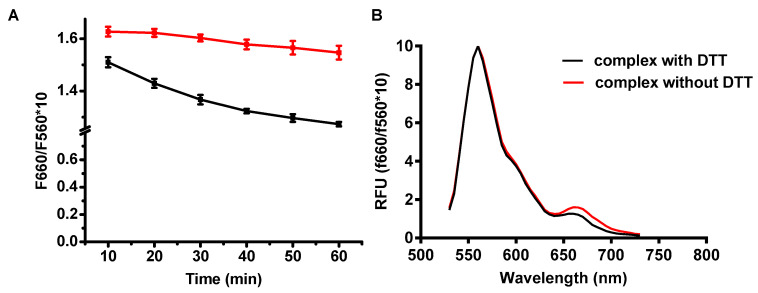
The measured FRET intensities of nanocomplex with or without DTT as a function of time (**A**) and full spectrum of complex with or without DTT when measured the FRET intensity (**B**).

A unique feature of intercellular environment is its high reduction potential due to the elevated GSH, which plays an important role in maintaining the reducing environment to prevent the excessive oxidation stress from normal activities such as electron transfer from respiration or photosynthesis [32]. Our polymer LBA-PDAPEG would be almost neutral after exposing it to a high reduction environment due to the lost functionalization of carboxylate group and amine group. Thus, the complex would be dissociated quickly and would release melittin accordingly. To evaluate how the complex responds to a high GSH environment, fluorescence resonance energy transfer (FRET) based nanocomplex was prepared as described, using Cy3 as energy donor and Cy5 as energy receptor. Excited at 530 nm of Cy3, the energy was transferred from PDAPEG-Cy3 (donor) to melittin-Cy5 (acceptor), which led to an increased emission intensity at 670 nm. A reducing environment, such as 10 mM DTT, was able to cleave carboxylate groups. The dissociation of neutral polymer and positive melittin led to a decreased energy transfer, which decreased the fluorescence intensity at 660 nm as shown in Figure 6. The fluorescence intensity was reduced by 20% after 50 min incubation with DTT, suggesting that the nanocomplex integrity was partially compromised by a high reducing environment.

### 2.4. Hemolytic Assay

**Figure 7:**
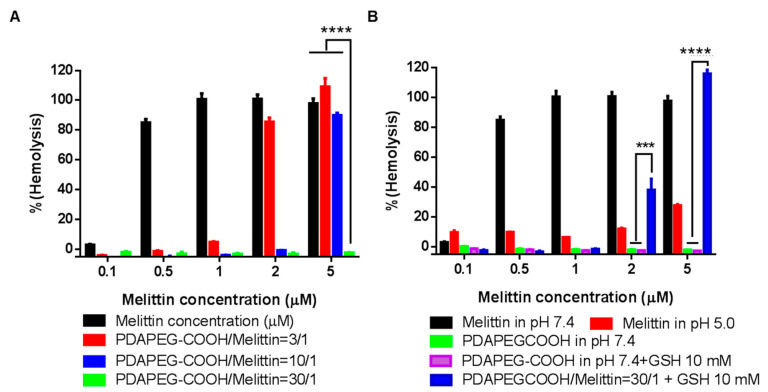
Hemolytic activity of melittin and nanocomplex. (**A**) The hemolytic activity of PDAPEG/melittin nanocomplexes of different polymer to melittin ratios. (**B**) The hemolytic activity of melittin, PDA-PEG, and PDAPEG/melittin nanocomplexes in different pH and reducing environments. *** *p* < 0.001, **** *p* < 0.0001.

To evaluate the safety of the polymer/melittin nanocomplex, hemolysis assay was adopted. Free melittin was able to lysis more than 80% of red blood cells (RBCs) at the concentration of 0.5 µM, whereas the hemolytic activity of the polymer/melittin nanocomplex decreased with the increase of polymer to melittin ratio (Figure 7A). No RBC lysis was observed for the nanocomplexes formed at the polymer/melittin ratio of 30 at the melittin concentration of 5 µM, indicating the excellent safety of the nanocomplexes. As expected, after the co-incubation with 10 mM GSH at 37 °C for 2 h, the nanocomplex solution lysed 40% and 100% of RBCs at 2 µM and 5 µM, respectively (Figure 7B). It is believed that the complex was dissociated upon the increased reducing environment and released the free melittin to form pores on the surface of RBCs and lysis them.

### 2.5. In vitro Cellular Uptake

**Figure 8:**
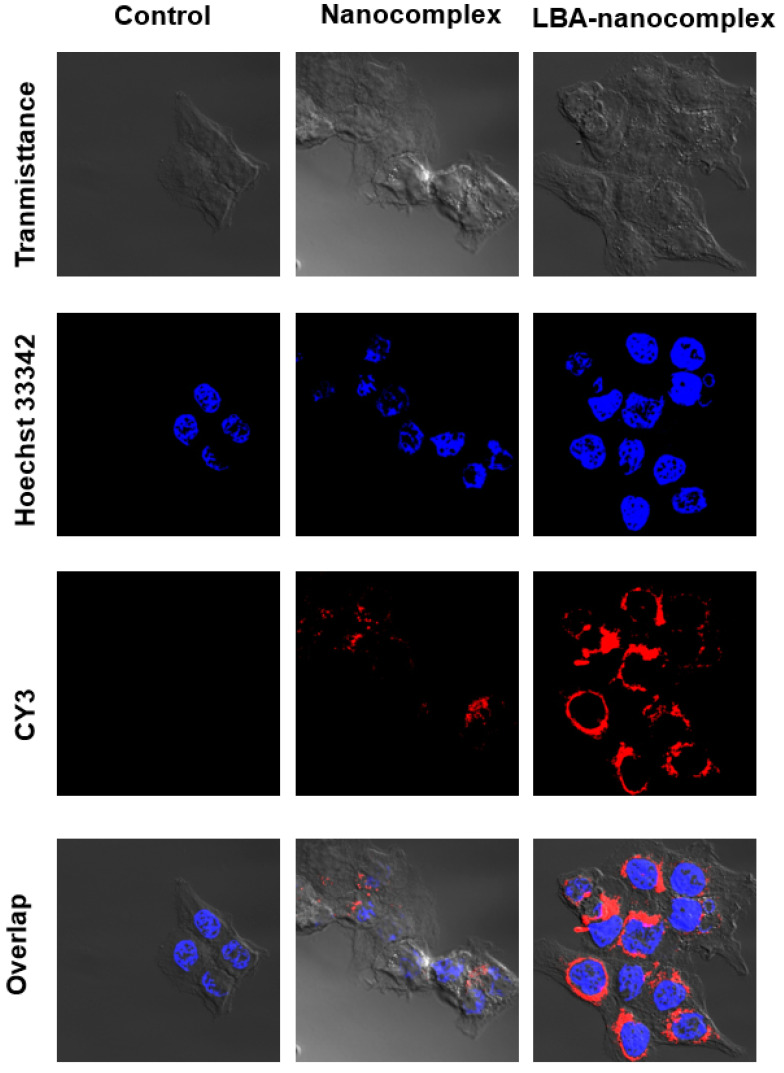
Confocal microscope images of HCT-116 colon cancer cells after incubating with control nanocomplex or LBA-nanocomplex for 3 h.

It has been reported that LBA can bind to cancer cells membrane overexpressed D-galactose receptors. To investigate the cellular uptake of LBA-nanocomplexes, confocal microcopy was applied. With a short term of incubation with HCT 116 cells, LBA-modified nanocomplexes showed a significantly increased uptake compared with the non-targeted group, as evidenced by the brighter red fluorescent (Figure 8). This data indicated that LBA could serve as a targeting moiety toward HCT 116 colon cells. A similar LBA based targeting effect was also observed in the MCF-7 cells (data not shown).

### 2.6. In vitro Anticancer Activities

**Figure 9:**
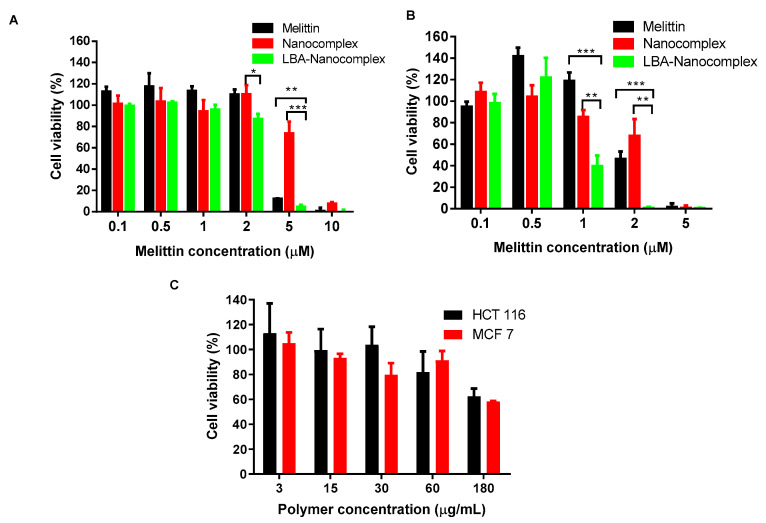
Cytotoxicity of melittin, control nanocomplex, and LBA-nanocomplex for various cancer cells. Cells were incubated with melittin nanocomplex and LBA-nanocomplex at melittin concentration from 0.1 to 10 µM for 24 h. Data represent mean ± SD, (**A**) HCT 116 cell; (**B**) MCF-7 cells; (**C**) Polymer cytotoxicity for HCT-116 and MCT-7 cells. * *p* < 0.05, ** *p* < 0.01, *** p < 0.001.

The cytotoxicity of nanocomplex was investigated toward various cancer cells, including HCT 116 and MCF-7 cells, as shown in Figure 9. Compared with free melittin, non-targeted nanocomplex showed a similar effect at low concentration but less effective at concentration higher than 1 µM in killing cancer cells. Remarkably, the LBA-nanocomplex showed an enhanced cytotoxicity compared to non-targeted nanocomplex and also significantly higher potency than free melittin at all concentrations. For HCT 116 cells, the IC 50 for nanocomplex was 4.15 µM and the LBA-nanocomplex was 1 µM. The targeting effect contributed a 76% decrease. Although melittin is more toxic toward MCF-7 cells, which killed slightly less than 90% of cells at the concentration of 5 µM, LBA-nanocomplex could almost eradicate all cancer cells at the same dose. Most melittin-loaded nanoparticles exhibit similar or lower potency than free melittin in killing cancer cells [21,33]. Based on our findings and the reported results from other research groups, we conclude that LBA-nanocomplex exhibited a better efficacy toward various cancer cells than free melittin mainly due to its cancer-targeting effect [34].

### 2.7. In Vivo Experiment

**Figure 10:**
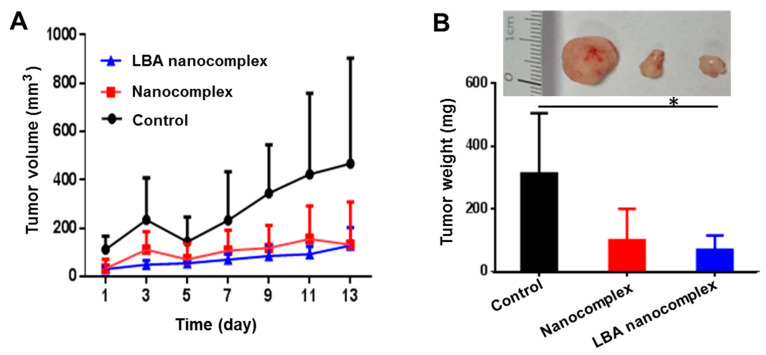
Tumor growth inhibitory effect of the LBA nanocomplex. Tumor growth profile (**A**) and tumor weight (**B**) after receiving different treatments. Representative tumor images of each group at the end of treatment (insert). * *p* < 0.05.

To assess the tumor growth inhibitory effect of the nanocomplex, the in vivo experiment was carried out in a xenograft HCT 116 colon tumor-bearing nude mouse model. Figure 10A revealed that polymer/melittin nanocomplexes remained active. Both the non-targeted and targeted nanocomplex can effectively inhibit the growth of tumors. After two weeks of treatment, the size of tumors in the nanocomplexes treated groups was around half of the tumor size in the control group. After the sacrifice of the animals, the tumors were isolated and weighed. Figure 10B indicated the tumor weight of LBA nanocomplex treated group was less than ¼ of that in the control group. Consequently, the LBA nanocomplex showed a significantly better tumor growth inhibitory effect than the control.

## 3. Conclusions

In this study, we successfully developed a polymer/melittin based nanocomplex to evaluate its safety and efficacy in cancer therapy. It was revealed that the nanocomplex can effectively deliver melittin into cancer cells and release its contents responding to high redox potential environment while protecting RBCs from the damage of free melittin. The LBA-nanocomplex is more effective in killing both HCT 116 colon cancer cells and MCF-7 breast cancer cells. Furthermore, the in vivo experiment proved that the LBA-nanocomplex can effectively kill cancer cells, thus resulting in significantly reduced tumor progression in a xenograft colon cancer mouse model. This redox sensitive polymer/melittin nanocomplexes will provide a new approach for melittin based cancer therapy.

## 4. Materials and Methods

### 4.1. Materials

Melittin was purchased from SERVA Electrophoresis GmbH (Heidelberg, Germany); 3-mercaptopropionic acid, aldrithiol-2, acryloyl chloride, poly(ethylene glycol)methacrylate (Mn = 360 Da), cysteamine hydrochloride, hydrochloride acid, trimethylamine, 2-(N-morpholino) ethanesulfonic acid (MES), 2,2-azobis(2-methylpropionitrile) (AIBN), lactobionic acid (LBA), N-hydroxysuccinimide (NHS), and cysteamine hydrochloride were purchased from Sigma-Aldrich (St. Louis, MO, USA). EDC (1-thyl-3-(3-(dimethylamino)propyl) carbodiimide) was purchased from TCI (TCI America, Domorah, PA, USA); 2,4,6-trinitrobenzene sulfonic acid was purchased form Life Technologies. Sodium dodecyl sulfate (SDS) was purchased from Bio-Rad Laboratories, Inc (Hercules, CA). HCT 116 human colorectal cancer cells and MCF-7 breast cancer cells were obtained from American Type Culture Collection (ATCC).

### 4.2. Preparation of Melittin Polymer

PDA (2(pyridine-2-yldisulfanyl)ethyl acrylate) was synthesized with using aldrithiol-2 and mercaptoethanol as described [23]. PDA was then copolymerized with poly (ethylene glycol) methacrylate at 1:1 ratio through AIBN initiated free radical polymerization to obtain poly[((2-(pyridin-2-yldisulfanyl)ethyl)acrylamide) co-[poly(ethylene glycol)]] (PDA-PEG).

In order to produce PDA-PEG polymer with different amounts of amine and carboxylate groups, cysteamine and 3-mercaptopropionic acid were conjugated to PDA-PEG at different ratios (amine/carboxylate, molar ratio) through the thiol-disulfide exchange reaction (Figure 2), and the resultant products were purified three times by precipitation in cold ether. Different feeding ratios between cysteamine and 3-mercaptopropionic acid yield polymers with different isoelectric points (IEP).

LBA functionalization was carried out through EDC/NHS reaction. LBA was first activated with EDC/NHS in 0.1M MES buffer at pH 6.0 for 30 min, and then polymer PDA-PEG with modification of amine groups dissolved in PBS 7.4 at 20 mg/mL was added. The mixture was allowed to react overnight at room temperature. Free LBA was removed by repeated dialysis using Spectra/Por^®^ dialysis tube (MWCO, 3.5 KDa) against dd H_2_O for 48 h. Purified polymer was freeze-dried and stored at −20 °C.

The pyridine concentration in the polymer was quantitatively determined by measuring the UV absorbance at 375 nm after the cleavage by DTT. The concentration of pyridine was calculated based on the extinction coefficient for pyridine-2-thione in DMSO at 375 nm (8080 M^−1^ cm^−1^). The molecular weight of polymers was determined by GPC Viscotek GPCmax VE 2001 GPC solvent/sample module equipped with Viscotek VE 3580 RI detector and 270 Dual Detector (Malvern Instruments, Worcestershire, United Kingdom) using THF as mobile phase, and the structure composition was determined by ^1^H-NMR. The success conjugation of 3-mercaptopropionic acid or cysteamine was confirmed by ^1^H-NMR (Bruker Avance III HD 300, Billerica, MA, USA) and with a UV-vis spectrometer (Beckman, DU650, Brea, CA, USA). The conjugation efficiency of LBA to PDA-PEG was quantified by a 2,4,6-trinitrobenzene sulfonic acid (TNBSA) assay using cysteamine hydrochloride as standard.

### 4.3. FRET Measurement

The donor fluorescence dye Cy3-NHS was chemically conjugated with PDA-PEG, and the receptor fluorescence dye Sulfo-Cy5-NHS was conjugated with melittin following our published method [29]. Cy3 labeled polymer and Cy5 labeled melittin were mixed to prepared nanocomplex. Samples were loaded into Corning^®^ 96 well black flat bottom plates and DTT was added to wells to cleave disulfide bonds. Samples were excited at 500 nm with the cutting off at 530 nm. The entire fluorescence spectrum (from 530 nm to 750 nm) of the complex treated prior to and after DTT was then recorded as a function of the time with the help of a SpectraMax M5 Multi-Mode Microplate Reader (Molecular Devices, San Jose, CA, USA).

### 4.4. Hemolysis Experiment

Rat whole blood was purchased from Bioreclamation (Hicksville, NY, USA). Red blood cells were washed with 150 mM NaCl and centrifuged at 300 rcf for 2 min until the supernatant became clear. The resulting red blood cells were suspended in PBS 7.4. Polymer complex or melittin was dispersed in different buffers at a series of concentrations from 0.1 µM to 5 µM. To mimic the physiological condition, nanocomplexes were dispersed in PBS 7.4 100 mM or PBS 5.0 100 mM. To mimic the intracellular condition, nanocomplexes were dispersed in 150 mM PBS + 10 mM GSH and incubated at 37 °C for 2 h. Melittin nanocomplex or melittin solution was incubated with above red blood cells for 1 h at 37 °C. The release of hemoglobin was quantified by measuring the absorbance at 405 nm of the supernatant in a microplate reader (ELX808, BioTek Instrument, Winooski, VT, USA) after centrifugation at 300 rcf. for 2 min. PBS and ddH_2_O treated RBCs were used as negative control and positive control, respectively. The degree of hemolytic was calculated as the following formula: hemolysis % = (Abs(test) − Abs(PBS negative control))/Abs(DI water positive control)) × 100%.

### 4.5. Cellular Uptake

HCT 116 colon cancer and MCF-7 breast cancer cells were cultured in Dulbecco’s modification of Eagle’s medium (Corning, Glendale, AZ, USA) supplemented with 10% FBS and 1% penicillin–streptomycin (Life Technology, Grand Island, NY, USA) at 37 °C in 5% humidified CO_2_ atmosphere. Cells were seeded in 35 mm Petri dishes with a density of 200,000 cells/petri dish and allowed to grow overnight. Both non-targeting and targeting nano-complexes were applied to cells at the Cy3 concentration of 2 µM for 3 h. Cells were washed three times with PBS and fixed with 4% formaldehyde, followed by staining with Hoechst 33,342 for 15 min. The cells were imaged with a confocal laser scanning microscope (LSM 700, Zeiss, Oberkochen, Germany).

### 4.6. In Vitro Cytotoxicity

HCT 116 and MCF-7 cells were seeded in 96-well plates at the density of 20,000 cells/well and incubated at 37 °C in 5% CO_2_ overnight. Melittin, non-targeted complex, or targeted complex were diluted with a complete medium at desired concentrations ranging from 0.1 to 10 µM. After 24 h of incubation, the media were replaced with 100 µL fresh media containing 1 mg/mL MTT reagent and incubated for 4 h. The formed MTT crystal was dissolved with a stop solution, and the final optical density of the medium was measured using a microplate reader (ELX808, BioTek Instrument, Inc., Winooski, VT, USA) at λ = 595 nm.

### 4.7. Tumor Growth Retardation Study

Female nude mice were purchased from the Jackson lab and randomly assigned into 3 groups (3 mice/group). All animal experiments were conducted in accordance with NIH regulations and approved by the Institutional Animal Care and Use Committee of the University of South Carolina (Protocol No. 100851, approval date: 23 September 2015). Total 5 million HCT 116 cells suspended in PBS were subcutaneously injected into mice. When the tumor volume reached about 30 mm^3^, the mice were anesthetized with 3% of isoflurane and treated with LBA melittin nanoparticle at 2 mg/kg via intratumoral injection. For control groups, the same amount of buffer used in preparing complex or non-targeted nanoparticles was employed.

### 4.8. Statistics Analysis

Data was expressed as mean ± standard deviation and was analyzed using Microsoft Excel and GraphPad Prism 6.0 (San Diego, CA, USA). Statistical analysis was performed using a one-way Analysis of Variation (ANOVA) among treatment groups and the control group. A *p* value < 0.05 was considered significant.
